# Influenza viruses circulation in a tertiary care children hospital in Rome: a comparison between 2022 and the previous 5 years

**DOI:** 10.1186/s13052-023-01519-3

**Published:** 2023-09-13

**Authors:** Stefania Ranno, Luana Coltella, Giulia Linardos, Velia Chiara Di Maio, Luna Colagrossi, Leonarda Gentile, Eugenia Galeno, Marta Luisa Ciofi degli Atti, Sebastian Cristaldi, Alberto Villani, Massimiliano Raponi, Carlo Federico Perno, Cristina Russo

**Affiliations:** 1 Unit of Microbiology and Diagnostic Immunology, Bambino Gesù Children’s Hospital, IRCCS, Piazza Sant’Onofrio, 4, Rome, 00165 Italy; 2 Epidemiology, Clinical Pathways and Clinical Risk, Medical Direction, Bambino Gesù Children’s Hospital, IRCCS, Rome, Italy; 3 Pediatric Unit, Pediatric Emergency Department, Bambino Gesù Children’s Hospital, IRCCS, Rome, Italy; 4 Medical Direction, Bambino Gesù Children’s Hospital, IRCCS, Rome, Italy

**Keywords:** Influenza, Year-round surveillance, Pediatric, Influenza-like Illness, Seasonality, Out-of-season circulation

## Abstract

**Background:**

Influenza surveillance aims to determine onset, duration and intensity of the seasonal Influence-like Illness (ILI); data collection begins in the week 42 of a year and ends in the week 17 of the following year. In this observational study, we report the experience of a tertiary care children hospital in Rome about Influenza viruses circulation during the calendar year 2022 (January-December) in comparison with the previous five years (2017–2021), with a special focus on the weeks 18–41, usually not under surveillance.

**Methods:**

This retrospective study involved 36782 respiratory samples referred to 21354 patients (pts), median age 2.63 years, admitted with respiratory symptoms at Bambino Gesù Children’s Hospital in the years 2017–2022. Respiratory viruses were detected by molecular Allplex™ Respiratory Panel Assays (Seegene, Korea).

**Results:**

Regarding the pre pandemic years, 2017–2019, distribution of Flu positive patients focused in the first weeks of the year (weeks 1–17). During the pandemic period, Flu was not detected. In 2022, 239 Flu viruses were identified: 37 FluA (weeks 1–17), 29 FluA (weeks 18–41) and 168 FluA and 5 FluB (weeks 42–52). For the year 2022, during the non-epidemic period, the number of Flu viruses detected corresponded to 12.1% of total Flu detected, respect to 0-1.7% for the previous five years (p < 0.001).

**Conclusions:**

When compared with pre SARS-CoV-2 pandemic years, our data show a significant increase in Influenza cases during weeks 18–41/2022 and reveal an unexpected summer circulation of these viruses: just weeks 26–30 showed to be influenza virus free. A national year-round Flu surveillance could be useful to understand if changing in influenza epidemiology is transitional or likely to persist in the following years.

**Supplementary Information:**

The online version contains supplementary material available at 10.1186/s13052-023-01519-3.

## Background

Influenza (Flu) is an acute respiratory infection caused by influenza viruses. It is a seasonal disease that, in the northern hemisphere, occurs during the winter period [1].

In humans it is carried on mainly by Influenza A, responsible both of pandemic and epidemic, and B viruses, responsible of epidemic [2].

Since 1952, global influenza surveillance has been conducted through WHO’s Global Influenza Surveillance and Response System (GISRS) by National Influenza Centers (NICs) [3]. Italian NIC is the “Istituto Superiore di Sanità (ISS)” that, with the contribution of the Ministry of Health, coordinates the national influenza surveillance net (InfluNet). Data collection on Influenza-like illness (ILI) begins in the week 42 of a year and ends in the week 17 of the following year (Flu season). Surveillance aims to determine the onset, duration and intensity of the seasonal epidemic (Epidemiological Surveillance) and to monitor the circulation of different types and subtypes of influenza viruses (Virological Surveillance) [4].

In the last decade, in Italy the greater number of Influenza-like syndromes was reported in the first weeks of each year. The only exception was the 2009–2010 season in which the FluA H1N1 virus pandemic moved up the incidence peak to the end of 2009, 45–46 weeks. During the first year of SARS-CoV-2 pandemic (overlapping with the 2020–2021 Flu season), ILIs were not reported and only in the following season (2021–2022) their incidence had a weak increase [5].

As known, one of SARS-CoV-2 pandemic consequences was the disappearance of respiratory viruses, with the only exception of Rhinoviruses [6–8]. During the year 2022, respiratory viruses came back and, with them, Influenza viruses [9].

In this observational study, we report the experience of a tertiary care children hospital in Rome about Influenza viruses circulation during the calendar year 2022 (January-December) in comparison with the previous five years (2017–2021), with a special focus on the weeks 18–41, usually not under surveillance.

## Methods

This is a retrospective analysis of patients admitted for suspected ILI at Bambino Gesù Children’s Hospital in Rome from 1st January 2017 to 31st December 2022. The study involved a total of 36782 respiratory samples, referred to 21354 patients, processed with a multiplex respiratory viruses assay, including Influenza A (subtype H1N1-pdm 2009, H3N2) and B viruses.

For each nasopharyngeal swab/aspirate collected, immediately or after storage at − 80 °C, identification of respiratory viruses was carried on by the multiplex one-step RT-PCR assay “Allplex^™^ Respiratory Panel Assays” on All-in-One Platform (Seegene, Korea). Nucleic acids were extracted using the STARMag Universal Cartridge kit (Seegene, Korea) on the automated Starlet platform and Real time PCR was performed on CFX96 (Bio Rad Laboratories). An internal control was included in each sample to check both extraction efficiency and PCR inhibition. In every run, a negative control, to monitor carry-over contamination, and a positive control, to check PCR reaction, were included. The results were analyzed automatically using Seegene software (Seegene Viewer V2.0).

According to datasheet indications for results interpretation, samples with a Cycle threshold (Ct) ≤ 42 were considered positive; samples without amplification or with a Ct > 42 were considered negative.

The whole workflow was performed according to manufacturer’s instructions.

When more than one positive test was available for a patient during the same clinical episode, only the first sample was included for the analysis.

Fisher’s exact test was used to analyze categorical variables. Statistical analyses were carried out using SPSS 19.0 (SPSS Inc., Chicago, IL, USA) and a p value of < 0.05 was considered to be statistically significant. Fisher’s exact test and Chi-squared test for trend were used to estimate significant changes among different years.

Since this is a retrospective observational study, results were collected in a completely anonymous and aggregate form, therefore the acquisition of consent as indicated by the Privacy Guarantor is not expected.

## Results

In the time interval under investigation 36782 respiratory samples, referred to 21354 patients (pts), median age 2.63 years (IQR: 0.57–7.84), were analyzed. (Table 1)”.

**Table 1:** Distribution of the study population (requests/samples, patients, median age) over the 6 years. IQR: Interquartile Range

Year	2017	2018	2019	2020	2021	2022	Total
Number of Requests/sample	5386	6697	7435	5777	5137	6350	36782
Number of Patients	3444	4044	4183	3422	2834	3427	21354
Median age yrs (IQR I-III)	1.73 (0.32–6.35)	1.95 (0.44–6.44)	2.45 (0.48–7.08)	3.41 (0.77–9.62)	2.85 (0.75–8.61)	3.71 (0.99–9.12)	2.63 (0.57–7.84)

From January to December 2022, were collected 6350 respiratory samples from 3427 patients (pts), median age 3.71 years (IQR: 0.99–9.12 IQR).

Among these, 237 respiratory samples referred to 237 patients (median age 4.4 years, IQR: 1.5–10.0) resulted positive for Influenza (Flu) viruses: 232 FluA, 3 FluB, 2 FluA plus FluB.

**Fig. 1:**
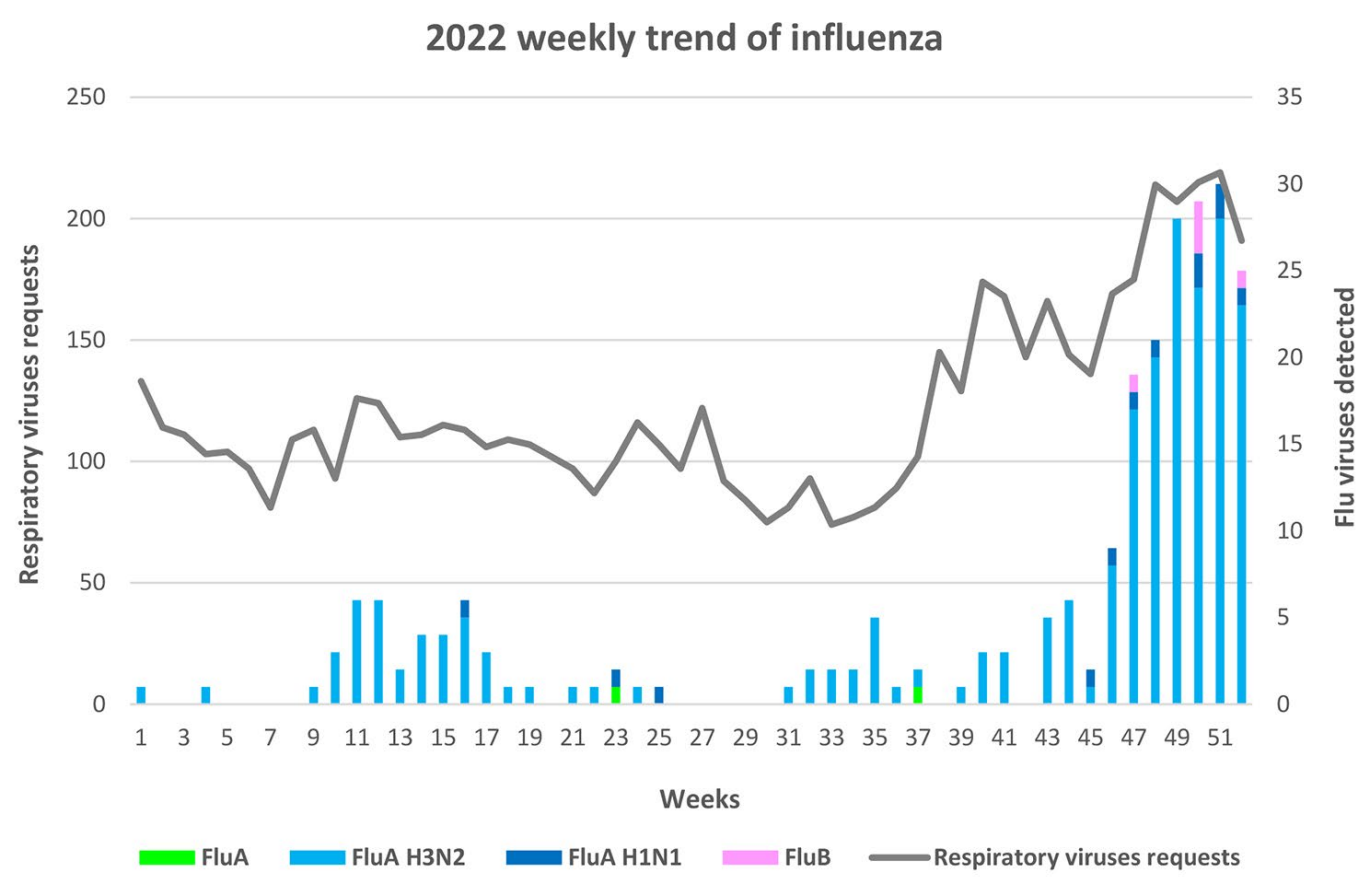
Trend of respiratory viruses requests (6350) and Influenza viruses detections (239) over the year 2022, including influenza virus type/subtype

Requests for suspected ILI and Flu positivities over the year 2022 are represented in Fig. 1.

FluA and FluB positivities were considered separately, so the number of influenza viruses detected (239) does not correspond to the positive patients (237), due to FluA plus FluB co-infection.

Circulation was further analyzed grouping requests and positive results according to the age of patients, range of age chosen were < 2 years, 2-5.99 years, 6-17.99 years and > = 18 years. No significant differences were noted since, in each group, positive samples/patients accounted for about 3–4% of requests.

Referring to the influenza season, 37 FluA viruses were detected in the weeks 2022, 1–17 (2021–2022 season), and 168 FluA and 5 FluB viruses were detected in the weeks 2022, 42–52 (2022–2023 season), accounting for 15.5% and 70.3% of total Flu viruses detected during the year 2022, respectively. FluB virus was detected starting from the week 2022, 47. During the period 2nd May − 16th October 2022 (weeks 2022, 18–41) 29 FluA viruses were also detected (12.1% of total Flu detected). Influenza viruses were not detected from 24th June to 1st August (weeks 2022, 26–30).

Regarding the whole study period, 2017–2022, distribution of Flu positive patients is shown in Table 2.

**Table 2:** Distribution of FluA and B positivities over the 6 years

Year	2017	2018	2019	2020	2021	2022
Requests	5386	6697	7435	5777	5137	6350
FluA positive (%)	231 (4.3)	198 (3.0)	287 (3.9)	193 (3.3)	2 (0.04)	234 (3.7)
FluB positive (%)	48 (0.9)	127 (1.9)	9 (0.1)	187 (3.2)	0	5 (0.1)
Flu positive* (%)	279 (5.2)	325 (4.9)	296 (4.0)	380 (6.5)	2 (0.04)	239 (3.8)
Flu positive pts	278	322	296	377	2	237

* FluA and FluB positivities were considered separately, so the number of influenza viruses detected (Flu positive) does not always correspond to the positive patients (Flu positive pts), due to cases of FluA plus FluB co-infection

Focusing on non-epidemic weeks (18–41), we have investigated the number of requests received for suspected ILI and the related Flu circulation for the six years. Results are reported in Table 3.

**Table 3:** Report of annual requests and FluA and B positivities with a focus in the weeks 18–41

Year	2017	2018	2019	2020	2021	2022
Requests for year	5386	6697	7435	5777	5137	6350
Flu positive for year	279	325	296	380	2	239
Requests weeks 18–41 (%)	1785 (33.1%)	2603 (38.9%)	2519 (33.8%)	1767 (30.6%)	2032 (39.6%)	2502 (39.4%)
FluA pos weeks 18–41 (week)	1 (41)	3 (18)	2 (18, 19)	0	0	29 (Fig. 1)
FluB pos weeks 18–41 (week)	1 (41)	2 (41)	3 (19, 38, 40)	0	0	0
Flu positive weeks 18–41 (%)	2 (0.7%)	5 (1.5%)	5 (1.7%)	0	0	29 (12.1%)

For the years 2017–2021, in these weeks, the number of requests for suspected ILI ranged from 30.6 to 39.6%, with a nearly absent Flu circulation (0-1.7% of Flu positive patients). In the same period of the year 2022, requests were 39.4% of total and Flu positive patients were 12.1%.

In 2022, a significant increase of prevalence of Flu positive cases was observed when compared with each previous year (p < 0.001).

**Fig. 2:**
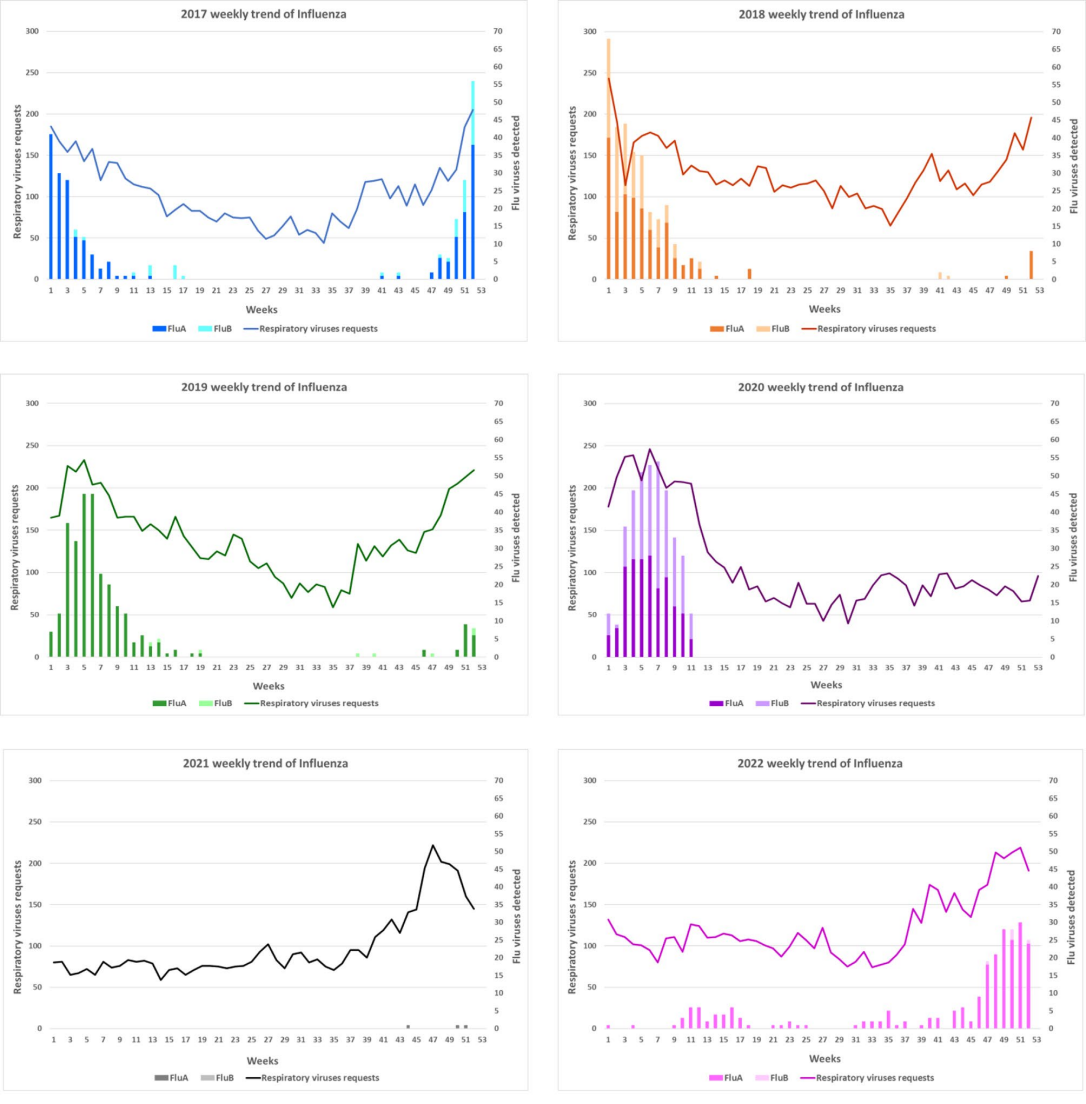
Trend of respiratory viruses requests and Influenza viruses detections over the years 2017–2022. x axis: weeks; y axis: number of respiratory viruses requests (line) and number of Flu positive patients (histograms)

Graphical distribution of respiratory viruses requests and Influenza viruses detections for each year of the study period is reported in Fig. 2.

## Discussion

For what concerns the epidemiology of the last six Flu seasons, our data are globally consistent with those reported in the National Epidemiological Report. For the 2017-18 season the ILI peak is reported in the second week of 2018, with an incidence level of about 15 cases per 1000 assisted. Both in the 2018–2019 and 2019–2020 seasons, it occurred approximately in the fifth week, with an incidence of about 14 and 13 cases per 1000 assisted, respectively. The incidence between 2020 and 2021 was almost zero, because the use of facial masks and lockdown, introduced for SARS-CoV-2 pandemic, reduced circulation of all those pathogens with airborne and droplet transmission. In the 2021–2022 season, two slight ILI peaks occurred approximately in the 52nd week of 2021 and in the 13th week of 2022, with an incidence of about 5 cases per 1000 assisted [5]. For the 2022–2023 season a peak in the 48th week of 2022 with an incidence of 16 cases per 1000 assisted [9] was reported, which indicates that the flu season began extremely earlier than previous years.

Aim of this study was to evaluate what happened in the period out of influenza virus circulation (weeks 18–41), normally not investigated by InfluNet.

For the years 2017–2021, in the non-epidemic weeks, we can appreciate a significant number of requests for suspected ILI (33–39%) each year and, as expected, a clear reduction of Flu circulation. The few positive patients were detected close to the end or the beginning of the previous or following flu epidemic season, respectively. Unexpectedly this was not observed for the year 2022, in which, in spite of a comparable number of requests (39%), the rate of influenza diagnosis was 12.2% (29 pts, p < 0.001) of total Flu positive patients detected (237 pts) over the year. In our population the first case of Influenza virus was detected even on August (week 31) and it was referred to Flu A (H3N2).

As reported in the National Virological Report for the year 2022 [10], our results confirm the prevalence of FluA (H3N2) (220/239 Flu viruses detected, 92%). Genetic characterization of FluA (H3N2) viruses circulating during the weak peak occurred in the weeks 2022, 10–16, presented a new phylogenetic makeup of their hemagglutinin respect to the 2021–2022 vaccine strain [11].

Influenza molecular surveillance plays a critical role to timely assess vaccine effectiveness and detect novel strains with potential impact on public health. Further analysis will be performed to obtain the genetic characterization of the strains circulating both in the non-epidemic period and in the weeks of the 2022–2023 season.

From an epidemiological point of view, our results about a Flu summer circulation are in agreement with those reported in a recent paper by Mellou K., in which an unusual out-of-season influenza activity in the summer of 2022 in Greek islands has been described [12].

Moreover, it is known that, in tropical climates, influenza viruses circulate throughout the year and no distinct seasonal patterns can be observed [13]. Thus, climate changes could be another factor to consider, assuming a stable change in flu seasonality.

## Conclusions

The main evidence emerging from this study was the unexpected circulation of influenza virus in the summer period of the year 2022. Surprisingly, only weeks 2022, 26–30, showed to be influenza virus free.

The debate remains open: did SARS-CoV-2 pandemic really change the influenza epidemiology, did we just describe a transitional phase after the absence of respiratory viruses in which they are resuming their usual place or did climate change definitively modify flu circulation?

A year-round flu surveillance could be useful to start having information to answer these questions.

### Electronic supplementary material

Below is the link to the electronic supplementary material.

Supplementary Material 1

## Data Availability

The datasets used and/or analysed during the current study are available from the corresponding author on reasonable request.

## List of Abbreviations

CT: Cycle threshold
Flu: Influenza
ILI: Influenza-like Illness
IQR: Interquartile Range
Pts: Patients
Yrs: Years
