# Supplementary material for: Influenza viruses circulation in a tertiary care children hospital in Rome: a comparison between 2022 and the previous 5 years
Source: Ital J Pediatr. 2023 Sep 13;49:121. doi: 10.1186/s13052-023-01519-3 (PMC10500714; doi:10.1186/s13052-023-01519-3)
Supplement: Supplementary file 1 — Supplementary Material 1 [file 13052_2023_1519_MOESM1_ESM.docx]

Unit of Microbiology and Diagnostic Immunology

Bambino Gesù Children Hospital, IRCCS

Rome, Italy

Professor Giovanni Corsello

Editor-in-Chief

*“Italian Journal of Pediatrics”*

Rome, 27^th^ July 2023

Dear Editor,

We would like to propose to your attention our original article entitled “*Influenza viruses circulation in a tertiary care children hospital in Rome: a comparison between 2022 and the previous 5 years”* by Stefania Ranno_,_ Luana Coltella, Giulia Linardos, Velia Chiara Di Maio, Luna Colagrossi, Leonarda Gentile, Eugenia Galeno, Marta Luisa Ciofi degli Atti, Sebastian Cristaldi, Alberto Villani, Massimiliano Raponi, Carlo Federico Perno and Cristina Russo, for potential publication in your Journal.

It is well known as respiratory viruses epidemiology has changed during the SARS-CoV-2 pandemic, in particular, Influenza (Flu) viruses have nearly disappeared for a year.

In this manuscript, we report Flu viruses circulation in a tertiary care children hospital in Rome, from 2017 to 2022. We focus our attention on the influenza non-epidemic period (weeks 18-41), during which, for the pre SARS-CoV-2 pandemic years, Flu circulation was nearly absent. In the same period, for the year 2022, the rate of influenza diagnosis was about 12%, revealing an unexpected Flu summer circulation.

We believe that our work provides an opportunity to draw attention to the circulation of influenza viruses during non-epidemic periods and contributes to the debate on the ongoing changes in the epidemiology of respiratory viruses.

Since this is a local surveillance observational study in a pediatric population, we think that *“Italian Journal of Pediatrics”* could be the appropriate journal to submit our work.

We confirm that this manuscript has not been published elsewhere and is not under consideration by another journal.

All authors have contributed significantly to the work, they have approved the manuscript and agree with its submission to “*Italian Journal of Pediatrics*”.

We hope that the information provided with this manuscript will encounter your editorial approval and that it might be appealing for the audience of your prestigious journal.

Thank you for your attention,

Best Regards

Luana Coltella

Microbiology Unit

Bambino Gesù Children’s Hospital

Corresponding author

[luana.coltella@opbg.net](mailto:luana.coltella@opbg.net)

Bambino Gesù Children’s Hospital

Piazza Sant’Onofrio, 4

00165 Rome, Italy

telephone number +360668594016

fax number +390668592218
